# Anti-Yeasts, Antioxidant and Healing Properties of Henna Pre-Treated by Moist Heat and Molecular Docking of Its Major Constituents, Chlorogenic and Ellagic Acids, with *Candida albicans* and *Geotrichum candidum* Proteins

**DOI:** 10.3390/life13091839

**Published:** 2023-08-30

**Authors:** Sulaiman A. Alsalamah, Mohammed Ibrahim Alghonaim, Mohammed Jusstaniah, Tarek M. Abdelghany

**Affiliations:** 1 Department of Biology, College of Science, Imam Mohammad Ibn Saud Islamic University, Riyadh 11623, Saudi Arabia; saalsalamah@imamu.edu.sa (S.A.A.); mialghonaim@imamu.edu.sa (M.I.A.); 2 University Medical Service Center, Building 70, King Abdulaziz University, Jeddah 21589, Saudi Arabia; mjusstanih@kau.edu.sa; 3 Botany and Microbiology Department, Faculty of Science, Al-Azhar University, Cairo 11725, Egypt

**Keywords:** anti-yeast, moist heat, *Lawsonia inermis*, healing, phenolic, flavonoid, molecular docking

## Abstract

*Lawsonia inermis*, known as henna, has traditionally been utilized in cosmetics and folk medicine because of their valuable health effects. A lack of information about the processes that increase or decrease release, as well as the biological activities of constituents of natural origin, is an important pharmacological problem. This investigation evaluates the influence of moist heat on the flavonoid and phenolic contents of henna powder and their biological activities. HPLC analysis reflected the existence of 20 and 19 compounds of flavonoids and phenolics in the extract of unpre-treated henna by moist heat (UPMH) and pre-treated henna by moist heat (PMH). Several compounds such as chlorogenic acid, ellagic acid, rutin, rosmarinic acid, kaempferol, and pyrocatechol occurred with high concentrations of 57,017.33, 25,821.09, 15,059.88, 6345.08, 1248.42, and 819.19 µg/mL UPMH while occurred with low concentrations of 44,286.51, 17,914.26, 3809.85, 5760.05, 49.01, and 0.0 µg/mL, respectively in PMH. *C. albicans*, *C. tropicalis*, and *G. candidum* were more affected by UPMH with inhibition zones of 30.17 ± 0.29, 27 ± 0.5, and 29 ± 1.5 mm than PMH with inhibition zones of 29 ± 0.5, 25.33 ± 0.58, and 24.17 ± 0.29 mm, respectively. UPMH henna exhibited less MIC and MFC against the tested yeasts than PMH. Moreover, UPMH henna showed good wound healing, where the rat of migration, wound closure %, and area difference % were 14.806 um, 74.938 um^2^, and 710.667% compared with PMH henna 11.360 um, 59.083 um^2^, 545.333%, respectively. Antioxidant activity of UPMH and PMH henna. Promising antioxidant activity was recorded for both UPMH or PMH henna with IC_50_ 5.46 µg/mL and 7.46 µg/mL, respectively. The docking interaction of chlorogenic acid and ellagic acid with the crystal structures of *G. candidum* (4ZZT) and *C. albicans* (4YDE) was examined. The biological screening demonstrated that the compounds had favorable docking results with particular proteins. Chlorogenic acid had robust behavior in the *G. candidum* (4ZZT) active pocket and displayed a docking score of −7.84379 Kcal/mol, higher than ellagic acid’s −6.18615 Kcal/mol.

## 1. Introduction

*Lawsonia inermis* (*Lythraceae* family) is commonly recognized as henna and is native to subtropical areas of North Africa and Asia. Traditionally, it has been utilized as a dandruff-fighting and a controller for fungi when functional to the hair, feet, and hands, besides coloring of skin, hair, and nails was attributed to henna [1,2]. Several pharmacological properties were associated with henna extract, such as alleviating and ameliorating wound healing, antifungal, antibacterial, antioxidant, nootropic, hepatoprotective, anti-ulcer, anti-cancer, anti-inflammatory, and anti-cancer activity. However, the leaves of this plant represent the most valuable part, but roots, stem, and bark have been utilized in ethno medicine conventional medicine for over nine centuries [3,4].

Species of *Candida* are pathogenic yeast forming mucocutaneous and systemic complaints in humans, particularly in diabetes patients, transplant recipients, xerostomia, malignancy, malnutrition, and lowly oral hygiene (immunocompromised patients) [5,6]. Several species of *Candida* become resistant to antifungal compounds [7]. Therefore, there is a requisite for novel compounds to fight pathogenic yeasts with greater efficiency and low toxicity. Yiğit [3] tested the paste of *L. inermis* extract against several clinical *Candida* isolates including *Candida albicans*, *C. parapsilosis*, *C. glabrata*, *C. tropicalis*, *C. krusei*, *C. kefyr* with different levels of inhibition, where the inhibition zone was more than 20 mm against 35.4% of the isolates, and was up to 15 mm against 38.0%, while the rest of isolates were resistance to the extract of *L. inermis*. As reported by Samadi et al. [8], henna extract can be applied to treat oral cavity infections resulting from *C. albicans* because of the promising anti-candidal activity of the extract, with 2.8 mg/ mL as the minimum inhibitory concentration. Besides *Candida* spp., the henna leaves extract reflected a fungicidal effect against filamentous fungi, including *Penicillium ochrochloron*, *P. funiculosum*, *Aspergillus flavus*, *A. ochraceus* due to the presence of apigenin 5-glucoside in the extract [9]. *Trichophyton* spp. *Curvularia* spp. and *Geotrichum* spp. were affected by henna leaf extract because of the existence of terpenes, aliphatic compounds, and flavonoids [10].

Different extraction solvents (chloroform, ethanol, and methanol) and methods (hot sequential extraction) were applied to evaluate the biological activities of *L. inermis* [11]. Despite the use of natural materials since the beginning of humanity, and even the demand for them is increasing daily, there is a big gap between how to apply, the correct extraction methods, and pre-treatments to evaluate its effects on the type and quantity of active ingredients. In the present investigation, moist heat was applied to the powder of henna before the extraction process and its biological activities.

Some plants, such as *Laurus nobilis*, were subjected to microwave, and high temperature of oven up to 120 °C [12]. Also, Al-Rajhi et al. [13] studied the effect of moist heat on the phenolic and flavonoid contents of *L. nobilis*, where moist heat induced the release of constituents and increased its biological activities. To date, numerous studies have reported the biological activities of henna using several extraction solvents, but few, if any, investigations have assessed the phytochemical characterization and biological effect of pre-treated henna by moist heat. Therefore, the current investigation aimed to study the effect of moist heat on henna before extraction, anti-yeast and antioxidant activity, and healing properties. Also, the molecular docking interaction of the most detected constituents of henna extract with some tested yeasts.

## 2. Materials and Methods

### 2.1. Chemical Used

Methanol, acetonitrile, and 2,2-diphenyl-1-picrylhydrazyl (DPPH) were obtained from Sigma-Aldrich (Steinheim, Germany). Potato dextrose agar (PDA) medium was obtained from Oxoid Ltd., Basingstoke, Hampshire, UK.

### 2.2. Henna Source and Its Pre-Treated with Moist Heat

Dried henna leaves were obtained from the company of Abnaa Sayed Elobied Agro-Export, P.O. Box 10725, Khartoum, Sudan. The plant was validated by Prof. Marei A. Hamed, Prof. of the plant. The sample of leaves was kept under herbarium number SH 4325 in the Faculty of Science, Al-Azhar University, Egypt. The leaves were ground by a mill, and then passed via a 40-mesh sieve. The powder of Sudanian Henna was used in the current investigation. Henna powder (250 g) was autoclaved for 10 min at 100 °C, then cooled at room temperature (25 °C) and became pretreated by moist heat (PMH). At the same time, 250 g of henna was kept without pre-treatment by moist heat (UPMH) at 30 °C for 10 min with a humidity of 32%. The PMH and UPMH henna powders were extracted by mixing with 600 mL of methanol on the magnetic stirrer for 12 h. For removing any remains of the powders, the mixture at 5000 rpm for 10 min was centrifuged. Via rotary evaporator, the supernatant was concentrated to get a known weight, followed by re-dissolved in 0.5 mL of dimethyl sulfoxide (DMSO) [13].

### 2.3. Assessment of Phenolic and Flavonoid Constituents by HPLC 

UPMH and PMH henna extract were subjected to HPLC (Agilent 1260 series) for phenolic and flavonoid constituents’ detection. The separation process was performed via Zorbax Eclipse Plus C8 column (4.6 mm × 250 mm i.d., 5 μm). The flow rate of the mobile phase (MP) of water (W) and acetonitrile containing 0.05% trifluoroacetic acid (A) was 0.9 mL/min. The MP was automated sequentially in a linear gradient in the flowing order: 0 min (82% W); 82% W from 0–1 min; 75% W from 1–11 min; 60% W from 11–18 min; 82% W from 18–24 min. The ultraviolet (UV) detector was adopted at 280 nm and 330 nm for phenolic and flavonoid constituents’ detection, respectively. The solution of tested samples was injected in volume 5 μL with the column maintained at 40 °C. The input data of standard molecules of phenolic and flavonoids was used for the quantitative determination of the extract’s compounds [14].

### 2.4. Anti-Yeast Activity of UPMH and PMH Henna Extracts

The anti-yeast activity of the henna extracts was assessed according to Al-Rajhi et al. [15] with some modification via cup-plate agar diffusion technique against *Candida albicans* (ATCC 10231), *Geotrichum candidum* (RCMB 027016), and *Candida tropicalis*. The tested yeasts were standardized (Corresponding to 0.5 McFarland scale), sowed in molten sterile Sabouraud dextrose agar medium, and poured into petri dishes. After solidification, via sterile cork borer (6 mm radius), four cups were cut and removed. Via automatic microlitre pipette, 100 µL of 20 µg/mL of each extract was injected in each cup, and then kept in the refrigerator at 4 °C for 30 min to allow the extract to diffuse through the agar layer. Followed by the incubation at 35 °C for 48 h. The visualized inhibition zones were recorded using a calibrated ruler in millimeters.

### 2.5. Evaluation of Minimum Inhibitory Concentration of UPMH and PMH Henna Extracts

The extract of henna, including the unpre-treated and pre-treated henna powders, was tested to detect minimum inhibitory concentration against tested yeasts. According to the CLSI M27-A3 standard manner. For each species of yeasts, a dilution was prepared in an equivalent to 0.5 McFarland. A dilution of each extract was prepared (1 mg/mL). Using 96-well plates, 100 µL of RPMI 1640 broth adjusted at pH 7 using a buffer of MOPS were transferred into each well. 100 μL of each extract was mixed with RPMI in wells of 1st column, and then serial dilution was performed. From the suspensions of tested yeast, 100 μL (0.5 McFarland) were added to each well, and wells without yeast cell suspensions were used as a negative control. Followed by incubation for one day at 34 ± 2 °C. The lowest concentration of extract that gave 50% declined growth was defined as MIC compared to the controls. The antifungal nystatin was used as a positive control, while the solvent of the extraction (methanol) was used as a negative control, respectively.

### 2.6. Estimation of Minimum Fetal Concentration (MFC) of UPMH and PMH Henna Extracts

To assess the MFC, 50 μL of the clear homogenized well suspension (devoid of visual growth) was cultivated using Sabouraud Dextrose Agar plates, followed by incubation for 48 h at 35 °C. The lowest dose of the extract affected growth inhibition (99.9%) compared to growth at control (without treatment) was MFC. The count of each yeast colony (CFU/mL) at different doses was compared with the count of each yeast colony at control (without treatment).

### 2.7. Antioxidant Activity of UPMH and PMH Henna Extracts

The 1,1-diphenyl-2-picrylhydrazyl free radical (DPPH), developed by Elansary et al. [9] with minor modifications, was used to study the antioxidant scavenging activity. Various dilutions (1 mL) containing different concentrations of the plant extracts were combined with 1 mL of 0.2 mM DPPH dissolved in methanolic. Using a Helios spectrophotometer (Unicam, Cambridge, UK), the absorbance at 520 nm was measured following a 30-min incubation time at 25 °C. The same process was used in a blank experiment to create a solution devoid of the tested plant extract, and the absorbance was recorded. The percent inhibition of each solution’s free radical-scavenging activity was then determined using the following equation:

$$\%\;inhibition=(\frac{Absorbance\;of\;blank-Absorbance\;of\;extract}{Absorbance\;of\;blank})\times 100$$

Antioxidant activity was itemized as IC_50_, which is the amount of the tested extracts needed to result in a 50% drop in the initial DPPH concentration.

### 2.8. Healing Properties of UPMH and PMH Henna Extracts

A multiwell plate was used for scratch wound examination. The plate was coated with an extracellular matrix substrate of 10 μg/mL fibronectin. Followed by incubation at 37 °C for 2 h. Then, the unbound extracellular matrix was removed and washed with phosphate-buffered saline. The growing cells from a dish containing tissue culture were detached with trypsin. The cells were developed on the scratch wound assay plate, followed by incubation to permit cells to spread and to obtain a confluent monolayer. The monolayer cell, including the confluent monolayer, was scraped using a pipette tip. Once scratched, slightly wash the monolayer of cells to remove separated cells. Then, replace with fresh medium containing tested extracts. The plate was incubated at 37 °C in the incubator of cell culture for 24–48 h. After the end of the incubation period, the cell monolayer was washed using phosphate-buffered saline. Then, the cells were fixed for 15 minutes using 3.7% paraformaldehyde. The cells were stained for 10 min using crystal violet (1% in ethanol). Then, the cell culture was examined using a phase-contrast microscope [16]. The following analysis was calculated according to the following equations:

$$Rat\;of\;migration\;(RM)=\frac{Wi-Wf}{t}\times 100$$

where, Wi = average of initial wound width (um), Wf = average of final wound width (um), t = time span of the assay in hours

$$Wound\;clouser\;\%=\frac{A_{t0}-A_{t∆t}}{A_{t0}}\times 100$$

where, A_t0_ = intial wound area, A_t∆t_ = wound area after n hours

Area difference %=intial area−final area

### 2.9. Molecular Docking Investigation

Docking studies were carried out using the MOE (Molecular Operating Environment) software 2019.0102 program.

Ligand preparation: Chemical structures of substrate molecules (chlorogenic acid and ellagic acid) were drawn using ChemDraw Ultra 15.0, and this structure was saved as MDL files (“.sdf”) for MOE to show. These structures were optimized by adding hydrogens, and energies were minimized with parameters (gradient: 0.05, Force Field: MMFF94X).

Preparation of receptor structure: The *G. candidum* and *C. albicans* models were predicted through homology modeling. The best model was selected for docking analysis. This model is subjected to 3D protonation and energy minimization using parameters (gradient: 0.05, Force Field: MMFF94X + Solvation). The minimized structure was used as the receptor protein for Docking. The protein molecules utilised throughout our investigation were obtained from Protein Data Bank (http://www.rcsb.org/pdb accessed on 23 September 2015) using PDB codes (4ZZT) and (4YDE), respectively for *G. candidum* and *C. albicans*.

Docking Run: The MOE docking program with default parameters was used to bind the selected ligands with receptor proteins and to find the correct conformation of the substrate. Free energy of binding of the ligand from a given pose was estimated by MOE London dG scoring function. The top five poses were determined using hydrogen bonds with lengths under 3.5 Å and binding free energies (S, kcal/mol) between substances and amino acids that are part of proteins. Additionally, the RMSD and RMSD-refine fields were used to compare the results pose-with-pose in the co-crystal ligand position and before and after amendment, respectively.

### 2.10. Statistical Analysis

Standard deviation was calculated from the average three replicates of the obtained results via Microsoft programs of Excel version 365 and SPSS v.25. For variance analysis, one-way ANOVA, besides the test of post hoc Tukey, were applied to values analysis with a parametric distribution. The confidence interval was set to 95%, and the border of the accepted error was set up to 5%.

## 3. Results and Discussion

### 3.1. Flavonoid and Phenolic Contents of Unpre-Treated and Pre-Treated Henna by Moist Heat

**Figure 1:**
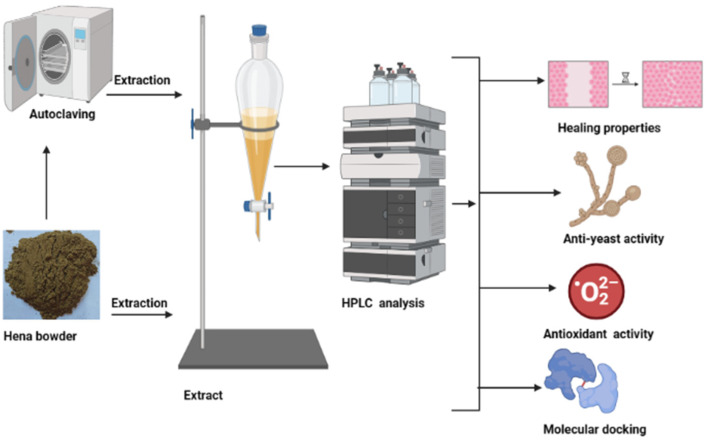
Designed tests for unpre-treated (UPMH) and pre-treated henna powder by moist heat (PMH) in an autoclave with different assessments, including HPLC analysis, healing properties, anti-yeast activity, antioxidant activity, and molecular docking of the main constituents of henna extract.

**Figure 2:**
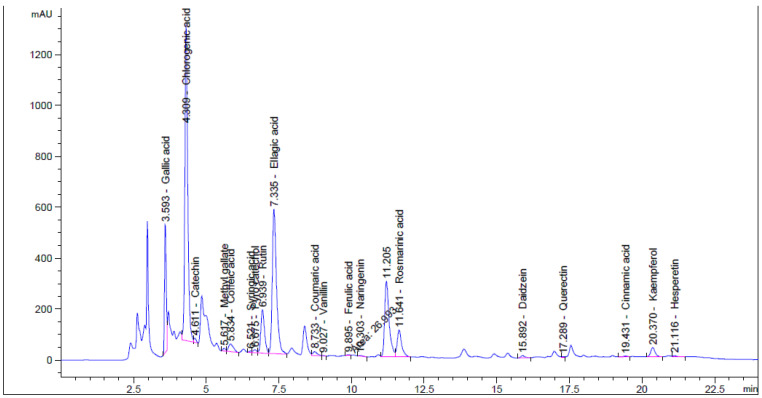
Phenolic and flavonoid compounds detection in UPMH henna extract by moist heat indicated by HPLC chromatogram.

**Figure 3:**
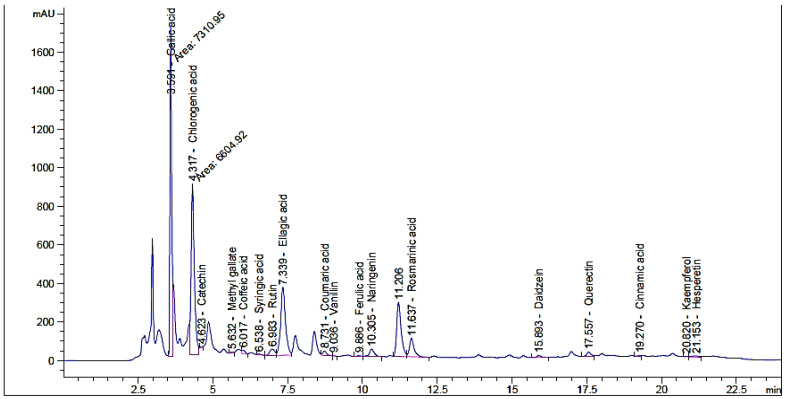
Phenolic and flavonoid compounds detection in PMH henna extract by moist heat indicated by HPLC chromatogram.

The effect of moist heat was evaluated on the contents of flavonoid and phenolic in Henna extract, as well as its anti-yeast properties and other biological activities were performed (Figure 1). From HPLC analysis, the extract of unpre-treated henna by moist heat (UPMH) reflected the existence of 20 compounds, while the extract of pre-treated henna by moist heat (PMH) reflected the existence of 19 compounds of flavonoids and phenolics with different retention times, area, area %, and concentrations (Figure 2 and Figure 3 and Table 1). Chlorogenic acid, ellagic acid, gallic acid, rosmarinic acid, and rutin represent the highest concentrations in both UPMH and PMH henna extracts. The results indicated that the concentrations of the most detected compounds decreased in PMH henna extract compared to UPMH. For example, the concentrations of chlorogenic acid, ellagic acid, rutin, rosmarinic acid, kaempferol, and pyrocatechol were 57,017.33, 25,821.09, 15,059.88, 6345.08, 1248.42, and 819.19 µg/mL in UPMH, while become 44,286.51, 17,914.26, 3809.85, 5760.05, 49.01, and 0.0 µg/mL in PMH henna extract On the other hand, quercetin, naringenin, gallic acid, and coumaric acid concentrations were 96.76, 133.45, 9349.90, and 270.56 µg/mL in UPMH while becoming 1269.47, 2146.89, 32,349.91, and 402.02 µg/mL in PMH henna extract, respectively. Some studies reported that the effect of moist heat induced the discharge of extract constituents, unlike the current study. For example, Juániz et al. [17] found that phenolic constituents of vegetables liberated more if they were pretreated with heat due to cell wall destruction by heat. The decrease of most compounds in pre-treated henna extract or missing compound (pyrocatechol) indicated that these compounds are unstable at high temperatures or heat-sensitive and, at the same time, may easily oxidize and transform into other compounds. This explanation may match with Khoddami et al. [14]. While the concentration of some compounds increased, it may be due to high temperature causing the destruction of plant cell walls, causing the release of internal compounds. Another clarification of this phenomenon is that some phenolic and flavonoid constituents exist in an insoluble form. Heat may break these constituents, leading to the discharge of these bound constituents.

Previously, Routray and Orsat [18] revealed that some phenolic ingredients were degraded when the natural plant extracts were exposed to the high power of the microwave. Dezashibi et al. [19] found that ultrasound of henna extract increments the phenolic constituents. Moreover, the content of total phenolic was affected by storage temperature and period. Other reports indicated that rises in temperature from 40 to 80 °C caused rises in total phenolic content [20]. Effects of boiling, microwaving, and boiling were reported on the content of ascorbic acid, β-carotene, and vitamin E in some plants, where visualized changes were observed [21].

### 3.2. Anti-Yeast Activity of Unpre-Treated and Pre-Treated Henna by Moist Heat

**Figure 4:**
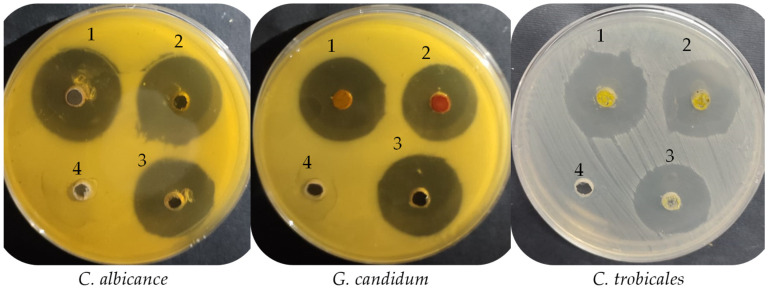
Anti-yeast activity of UPMH (1), PMH (2) Henna extracts, positive control (3) and negative control (4) against *C. albicance*, *G. candidum* and *C. trobicales*.

From the anti-yeast activity experiment, the extract of UPMH henna reflected significantly more inhibitory action with inhibition zones, 30.17 ± 0.29, 27 ± 0.5, and 29 ± 1.5 mm against *C. albicans*, *C. tropicalis*, and *G. candidum* compared with extract of PMH henna with inhibition zones, 29 ± 0.5, 25.33 ± 0.58, and 24.17 ± 0.29 mm, respectively (Table 2 and Figure 4). Moreover, the MIC and MFC of the extract of UPMH henna were less than the extract of PMH henna against all tested yeasts but with different degrees of sensitivity. Both types of extracts exhibited the highest anti-yeast activity than standard antifungal agents. According to the MIC and MFC, *G. candidum* was more sensitive, followed by *C. albicans* and *C. tropicalis*. The calculated index of MFC/MIC (≤2) indicated the cidal properties of both extracts. *L. inermis* showed anti-yeast activity but with different levels of inhibition depending on several factors, such as solvent extract, as mentioned previously by Suleiman and Mohamed [22], where the extraction by ethanol exhibited similar activity to nystatin, but the petroleum ether extract reflected more activity. Also, differences between the activities may be due to the region, the plant’s development, climatic changes, soil fertilizers, and the tested fungal population. Petroleum ether and ethanol extract of henna at 10 mg/mL demonstrated 26.3- and 25.3-mm inhibition zones against *Saccharomyces cerevisiae* 22.7-and-17 mm inhibition zones, respectively, against *C. albicans.* The inhibitory potential of the extract by the two types of solvents was more than that observed by the nystatin but did not give antifungal action against *Pichia fabianii* [22]. Kouadri [23] recorded strong anti-*C. albicans* activity of Saudi henna with an inhibition zone of 26 mm with a low MIC of 3.12 mg/mL due to several biologically active constituents. In vivo study, the henna (4%) was formulated as a vaginal cream, which showed promising management for infection caused by *C. albicans* in female rats. Moreover, it gave a similar effect to the antifungal agent (clotrimazole) [24].

### 3.3. Healing Properties of Unpre-Treated and Pre-Treated Henna by Moist Heat

**Figure 5:**
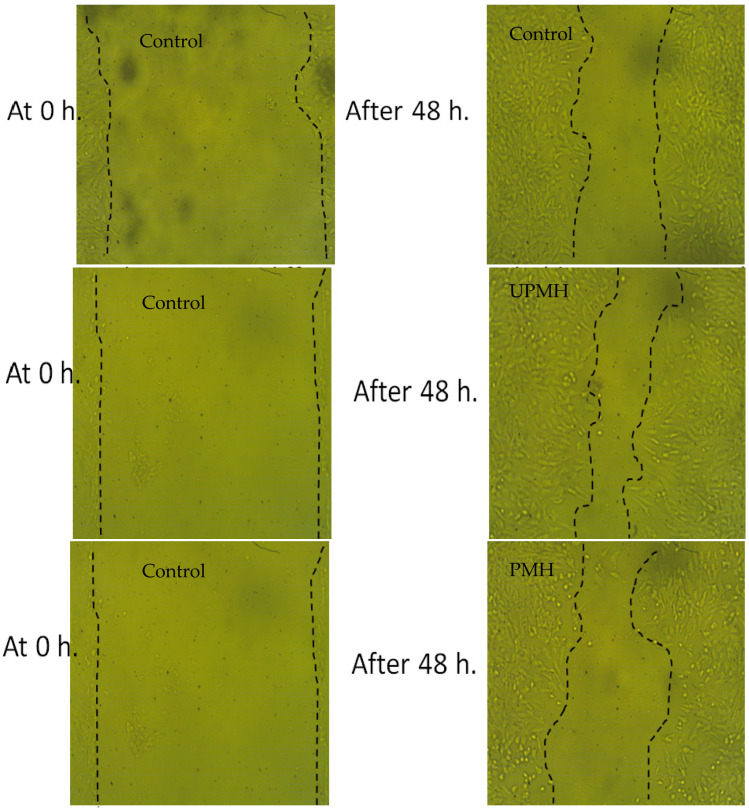
Images of scratch test illustrated the effect of UPMH and PMH henna extract by moist heat on the wounding area at 0 and 48 h.

There is a difference between the healing properties of the extract of UPMH and PMH henna, where the extract of UPMH henna provides reliable healing compared to the extract of PMH henna and control (cells without treatment) (Table 3 and Figure 5). The greatest common data resulting from the assessment of wound healing is the gap closure rate, which determines the rapidity of the cells’ collective motion. Rat of migration (RM), wound closure % and area difference % were 14.806 um, 74.938 um^2^, and 710.667% using extract of UPMH henna; 11.360 um, 59.083 um^2^, 545.333% using extract of PMH henna, compared to control cells, 11.554 um, 58.903 um^2^, and 554.667%, respectively with significant differs (Table 3). Daemi et al. [25] demonstrated that the healing process was accelerated by henna via minimizing tissue inflammation and increasing the uptake of glucose. An ointment containing henna extract shows a promising effect in managing episiotomy wounds [26]. El Massoudi et al. [27] explained the healing properties of henna. They mentioned that henna is richneed with different active molecules such as flavonoids, saponins, polyphenols, and others, which are vital in lowering oxidative stress and accelerating wound healing.

### 3.4. Antioxidant Properties of Unpre-Treated and Pre-Treated Henna by Moist Heat

The antioxidant activity of UPMH *and* PMH *henna* by moist heat was recorded in Table 4, compared with standard (Ascorbic acid). Generally, henna extract of either UPMH or PMH exhibited promising DPPH scavenging %. However, the extract of UPMH henna reflected more activity than the extract of PMH henna with IC_50_ 5.46 µg/mL and 7.46 µg/mL, respectively, compared with the IC_50_ value of ascorbic acid 2.52 µg/mL. The low activity of antioxidants for the PMH henna may be due to a low concentration of active compounds, as mentioned in HPLC analysis, due to exposure to moist heat. From Table 4, the antioxidant potential increments with the increasing dosage of the tested extracts with concentration-dependent liner. Réblov [28] studied the antioxidant activity of several phenolic acids under stress of temperature. For instance, vanillic acid was effective at 90 °C, while gallic and caffeic acids presented antioxidant potential at 150 °C. In in vivo investigation, henna protected them from oxidative stress and possessed hepatoprotective properties in Wistar rats [29]. Çubukçu et al. [30] reported that treatment by heat had harmful effects on the antioxidant activities of some plants, such as garlic and onion, while freezing enhanced the antioxidant properties of garlic and had negative effects on onion.

### 3.5. Molecular Docking of Chlorogenic Acid and Ellagic Acid with 4ZZT Protein of G. candidum and 4YDE Protein of C. albicans

In the current decade, molecular docking has attracted the attention of several investigators in drug design, development, and discovery. Structure-based computer modeling of ligand-receptor interactions is widely used in modern drug development. To identify conformational changes that vary with the environment and to characterize the interaction of the molecule with the protein with which it interacts inside the body, molecular docking calculations are commonly used in structure-based drug design investigations.

Chlorogenic and ellagic acids were docked using MOE (Molecular Operating Environment) in a vital trial to get insight into the potential pathways through which these compounds exert their antibacterial action. Both target proteins interact effectively with inhibitor compounds. The docking results of compounds’ interaction with the crystal structures of *G. candidum* (4ZZT) and *C. albicans* (4YDE) (4YDE code of the farnesyltransferase enzyme, and 4ZZT code of the enzyme cellobiohydrolase enzyme, which represent one of the virulence factors in yeast cells) revealed that chlorogenic acid was found the most promising than ellagic acid. Chlorogenic acid revealed substantial binding interactions within the (4ZZT) active site with a binding score of −7.84379 Kcal mol^−1^ according to five conventional hydrogen bonds between GLU 217 and O 19 with 2.80 Å, GLN 175 and O 17 with 2.97 Å, HIS 228 and O 19 with 3.24 Å, TRP 380 and C 1 with 3.19 Å, and TRP 371 and 6-ring with 3.61 Å. Also, ellagic acid outlined four significant interactions with active site residues of *G. candidum* (4ZZT) between GLU 217 and O 20 with 2.67 Å, THR 226 and O 22 with 3.20 Å, ARG 399 and O 24 with 3.08 Å, and ARG 251 and 6-ring with 4.09 Å.

**Figure 6:**
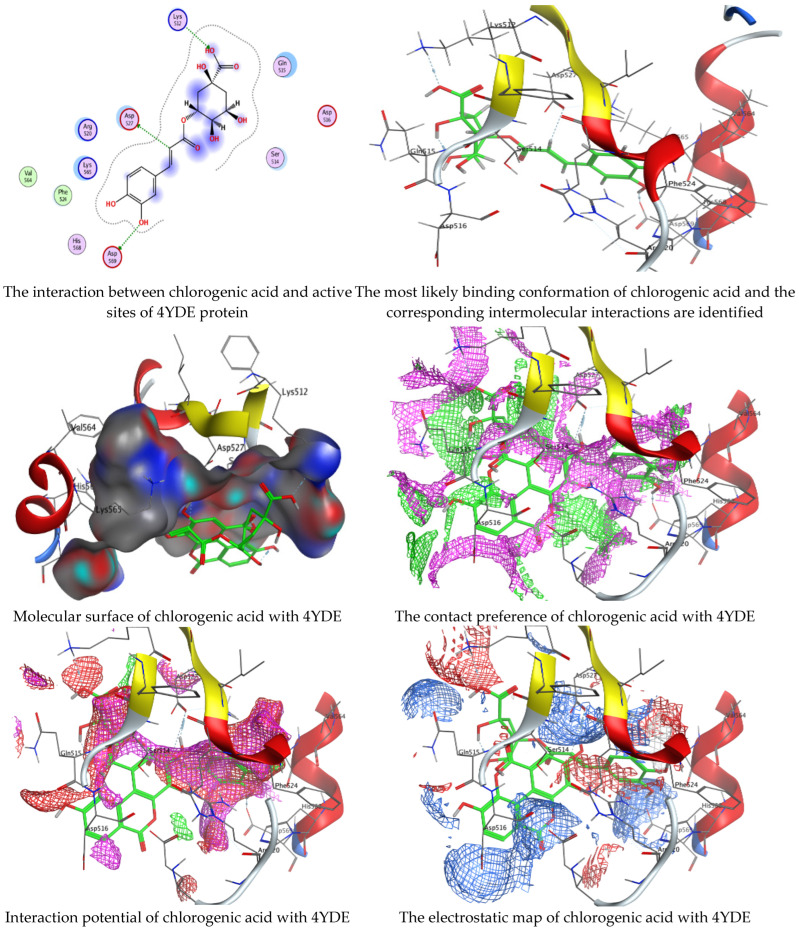
Molecular docking process of chlorogenic acid with the crystal structure of *C. albicans* (4YDE).

**Figure 7:**
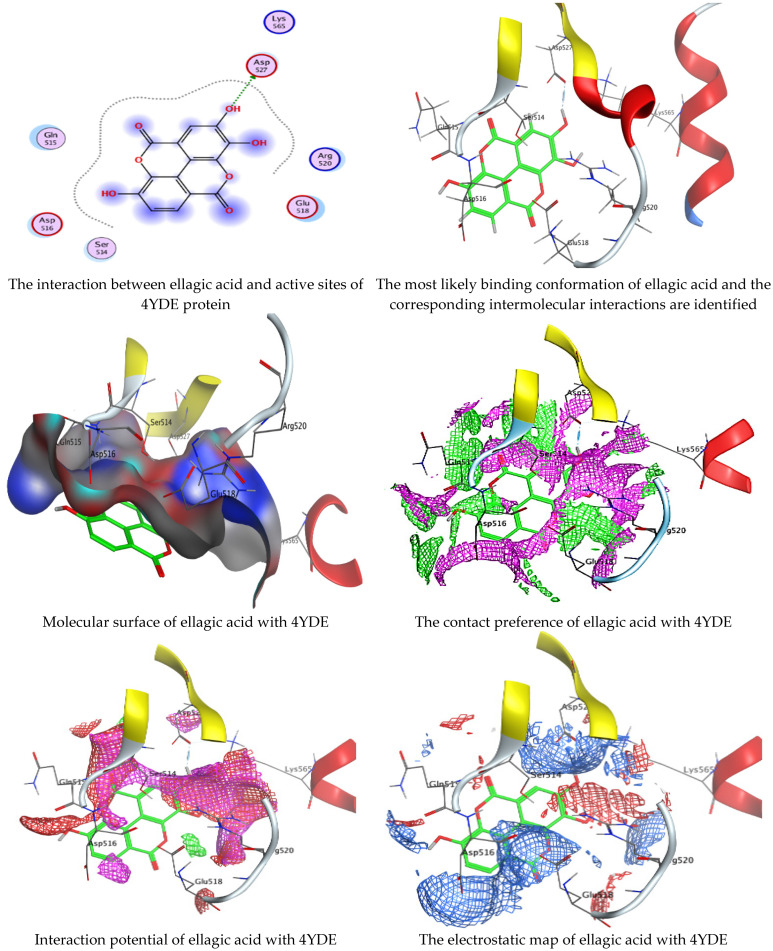
Molecular docking process of ellagic acid with the crystal structure of *C. albicans* (4YDE).

**Figure 8:**
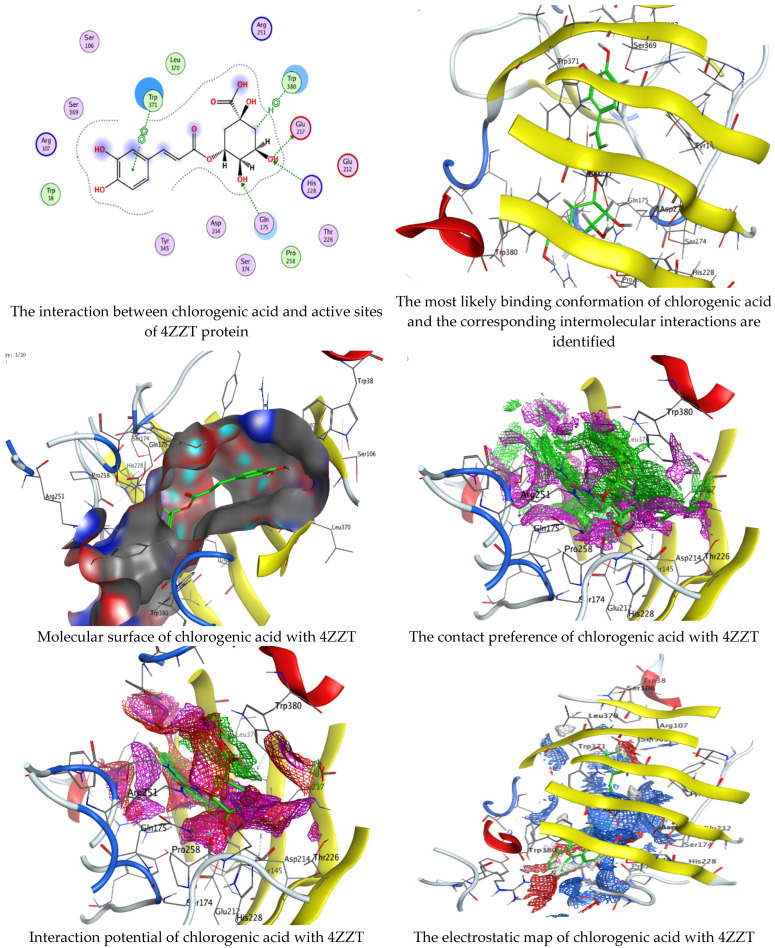
Molecular docking process of chlorogenic acid with the crystal structure of *G. candidum* (4ZZT).

**Figure 9:**
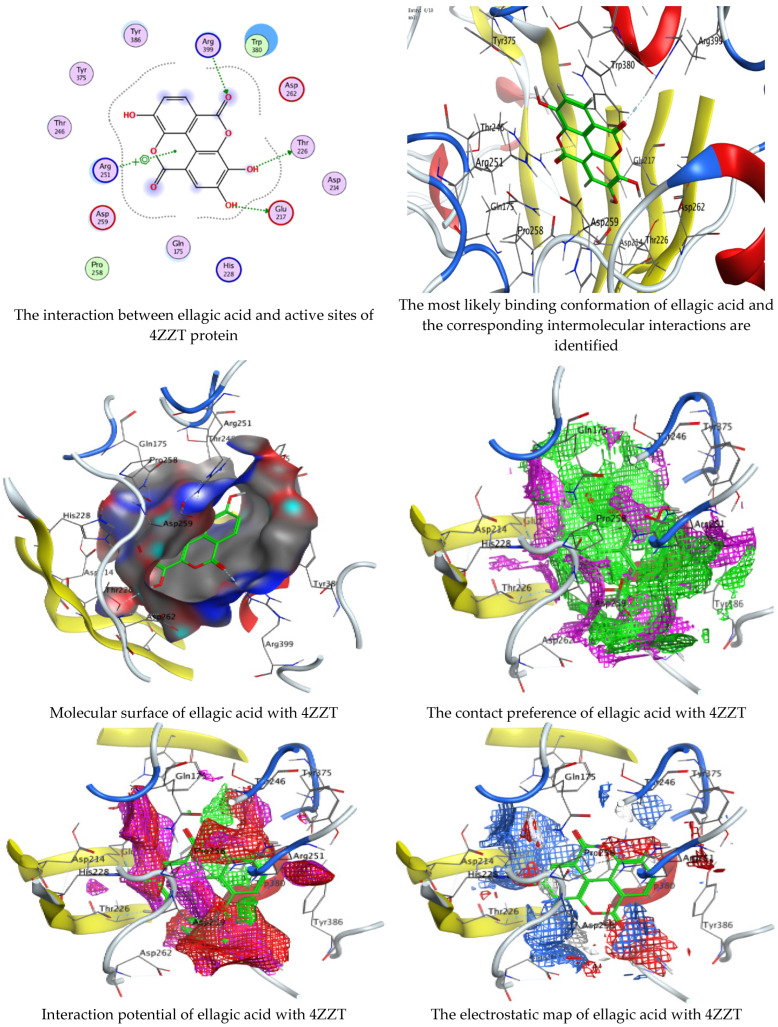
Molecular docking process of ellagic acid with the crystal structure of *G. candidum* (4ZZT).

Similarly, ranked the inhibitor compounds with *C. albicans* (4YDE) showed similar behavior and adopted direct H-donor and H-acceptor bonds with active site residues. Chlorogenic acid highlighted a binding score of −5.69876 Kcal mol^−1^, and exhibited several key interactions with (4YDE) with ASP 527, ASP 569, and LYS 512 via C 26, O 40, and O 23, respectively. While ellagic acid had a binding score of −4.5145 Kcal mol^−1^, and only one donor interaction between the O 20 atom and the ASP 527 amino acid residue, it demonstrated reduced effectiveness to (4YDE). The 2D and 3D docking interactions are given in Figure 6, Figure 7, Figure 8 and Figure 9, and the obtained results are shown in Table 5, Table 6, Table 7 and Table 8. Several reports confirmed the biological activities of drugs theoretically via molecular docking interaction [31,32,33,34,35,36]. The most potent constituent in henna (3a, 4a-Dihydroxy-a-tetralone) was docked with sterol 14-demethylase protein of *Staphylococcus aureus*, which reflected a docking score of −7.196 kcal mol^−1^ [37]. Alteration of yeast cells from the yeast shape to filamentous shape is linked with pathogenicity [38]. Therefore, the inhibitors of these enzymes (4YDE and 4ZZT) are potentially therapeutic against infection caused by yeasts.

## 4. Conclusions

The obtained results indicated that pre-treated henna powder with moist heat provided undesirable findings, where several phenolic and flavonoid compounds were decreased as exposure to moist heat. Moreover, the anti-yeast, antioxidant, and healing properties of un-pretreated henna were acceptable compared to pretreated henna by moist heat. In the search for new bioactive analogues, chlorogenic acid and ellagic acid exhibited good potential for *G. candidum* and *C. albicans* inhibition. According to docking data, chlorogenic acid performed the best interaction with the crystal structure of *G. candidum* (4ZZT), and the results can be utilized to guide future experimental studies. This investigation recommended the application of henna without any cocked process.

## Figures and Tables

**Table 1:** Identified phenolic and flavonoid compounds in unpre-treated (UPMH) and pre-treated (PMH) henna extracts by moist heat.

Compound	UPMH Henna Extract				PMH Henna Extract			
	Retention Time	Area	Area (%)	Conc. (µg/mL)	Retention Time	Area	Area (%)	Conc. (µg/mL)
Gallic acid	3.593	2113.04	8.9652	9349.90	3.591	7310.95	30.9495	32,349.91
Chlorogenic acid	4.309	8503.60	36.0792	57,017.33	4.317	6604.92	27.9606	44,286.51
Catechin	4.611	91.37	0.3877	1049.75	4.623	36.99	0.1566	424.95
Methyl gallate	5.617	67.04	0.2844	173.53	5.632	28.98	0.1227	75.00
Caffeic acid	5.834	358.26	1.5200	1469.54	6.017	103.97	0.4401	426.45
Syringic acid	6.521	35.46	0.1504	138.64	6.538	33.00	0.1397	129.02
Pyrocatechol	6.675	100.78	0.4276	819.19	6.652	0.00	0.00	0.00
Rutin	6.939	1493.20	6.3354	15,059.88	6.983	377.75	1.5991	3809.85
Ellagic acid	7.335	5712.41	24.2367	25,821.09	7.339	3963.18	16.7774	17,914.26
Coumaric acid	8.733	145.80	145.7976	270.56	8.731	216.64	0.9171	402.02
Vanillin	9.027	4.98	4.97855	9.41	9.036	4.57	0.0193	8.63
Ferulic acid	9.895	26.99	0.1145	82.29	9.886	41.57	0.1760	126.72
Naringenin	10.303	27.96	0.1186	133.45	10.305	449.88	1.9045	2146.89
Rosmarinic acid	11.641	1155.13	4.9010	6345.08	11.637	1048.63	4.4392	5760.05
Daidzein	15.892	86.45	86.4480	251.57	15.893	113.65	0.4811	330.74
Quercetin	17.289	15.82	0.0671	96.76	17.557	207.59	0.8788	1269.47
Cinnamic acid	19.431	38.59	0.1637	35.39	19.270	41.77	0.1768	35.30
Kaempferol	20.370	373.40	1.5843	1248.42	20.820	14.66	0.0621	49.01
Hesperetin	21.116	44.49	0.1888	118.00	21.153	122.14	0.5171	323.97

**Table 2:** Anti-yeast activity, MIC, MFC, and MFC/MIC index of UPMH, PMH henna extracts, positive control (Nystatin) and negative control (solvent used).

	Mean Inhibition Zone (mm)				MIC (µg/mL)		MFC (µg/mL)		MFC/MIC Index	
	UPMH	PMH	+ve C	−ve C	UPMH	PMH	UPMH	PMH	UPMH	PMH
*C. albicans*	30.17 ± 0.29 ^a^	29.00 ± 0.50 ^b^	26.0 ± 1.32 ^c^	0.0	15.63 ± 0.09 ^a^	15.64 ± 0.07 ^b^	31.23 ± 0.03 ^a^	31.25 ± 0.25 ^a^	1.99	1.99
*C. tropicalis*	27.0 ± 0.50 ^a^	25.33 ± 0.58 ^b^	25.17 ± 0.76 ^b^	0.0	62.50 ± 1.00 ^a^	125.33 ± 1.53 ^b^	125 ± 3.0 ^a^	249.67 ± 1.53 ^b^	2.0	1.99
*G. candidum*	29.0 ± 1.50 ^a^	24.17 ± 0.29 ^b^	27.0 ± 1.00 ^c^	0.0	7.83 ± 0.35 ^a^	15.62 ± 0.02 ^b^	7.8 ± 0.20 ^a^	15.62 ± 0.04 ^b^	0.99	1.0

Different higher letters for each species within a row (between UMH, MH, and +ve C in case inhibition zone or between UMH and MH in case MIC or between UMH and MH in case MFC) reveal significant differences (*p* ≤ 0.05).

**Table 3:** Healing properties of the extract of UPMH and PMH henna extract.

Treatment	At 0 h		At 24 h		At 48 h		RM um	Wound Closure % um^2^	Area Difference %
	Area	Width	Area	Width	Area	Width			
Control (without treatment)	885	884.081	737	736.024	381	380.021	11.554 ^a^	58.903 ^a^	554.667 ^a^
	937	936.009	737	736.000	377	376.021			
	959	958.052	741	740.219	361	360.355			
	945	944.008	837	836.038	337	336.095			
	959	958.000	849	848.021	413	412.000			
	965	964.000	843	842.086	453	452.004			
	Mean								
	941.667	940.692	790.667	789.731	387	386.083			
Extract of UPMH henna	931	930.002	813	812.089	249	248.008	14.806 ^b^	74.938 ^b^	710.667 ^b^
	953	952.034	819	818.002	281	280.007			
	945	944.172	823	822.01	249	248.129			
	943	942.034	837	836.117	183	182.176			
	971	970.132	749	748	229	228.000			
	947	946.008	763	762.042	235	234.034			
	Mean								
	948.333	947.397	800.667	799.71	237.667	236.726			
Extract of PMH henna	913	912.020	845	844.009	305	304.105	11.360 ^c^	59.083 ^c^	545.333 ^c^
	923	922.020	887	886.009	255	254.031			
	919	918.035	839	838.01	337	336.381			
	947	946.002	885	884.274	447	446.000			
	917	916.020	885	884.081	479	478.004			
	919	918.002	847	846.002	443	442.018			
	Mean								
	923	922.017	864.667	863.731	377.667	376.757			

Higher letters for the treatments within a column reveal significant differences (*p* ≤ 0.05).

**Table 4:** Antioxidant activity of unpre-treated (UPMH), pre-treated (PMH) henna extract by moist heat and ascorbic acid.

Concentration (µg/mL)	DPPH Scavenging %		
	UPMH	PMH	Ascorbic Acid
1000	98.5 ^a^	97.7 ^a^	99.3 ^ab^
500	95.0 ^a^	94.5 ^a^	96.3 ^b^
250	90.8 ^a^	90.1 ^a^	94.8 ^b^
125	83.2 ^a^	82.6 ^a^	91.9 ^b^
62.50	75.6 ^a^	74.4 ^a^	84.2 ^b^
31.25	69.1 ^a^	66.5 ^b^	76.1 ^c^
15.63	61.7 ^a^	59.3 ^b^	67.6 ^c^
7.81	53.3 ^a^	50.9 ^b^	60.4 ^c^
3.90	45.5 ^a^	41.5 ^b^	52.1 ^c^
1.95	37.8 ^a^	33.2 ^b^	43.7 ^c^
0.0	0.0	0.0	0.0
IC_50_	5.46 µg/mL	7.46 µg/mL	2.52 µg/mL

Different higher letters at each concentration within a row (between UPMH, PMH, and ascorbic acid) reveal significant differences (*p* ≤ 0.05).

**Table 5:** Docking scores and energies of chlorogenic acid and ellagic acid with the crystal structure of *C. albicans* (4YDE).

Mol	S	Rmsd_Refine	E_Conf	E_Place	E_Score1	E_Refine	E_Score2
Chlorogenic acid	−5.69876	1.6749115	−2.57228	−41.8448	−11.4368	−30.2837	−5.69876
Chlorogenic acid	−5.65288	1.729309	−2.45688	−57.55	−12.6225	−28.5364	−5.65288
Chlorogenic acid	−5.58586	1.9682481	14.59859	−48.7804	−12.7552	−28.076	−5.58586
Chlorogenic acid	−5.44998	1.4506954	9.080463	−45.4938	−11.5779	−26.7653	−5.44998
Chlorogenic acid	−5.41043	2.7580004	3.998731	−46.4482	−12.3195	−27.2703	−5.41043
Ellagic acid	−4.5145	4.868453	14.66806	−16.8034	−6.74256	−25.2636	−4.5145
Ellagic acid	−4.23481	3.9006364	16.43244	−48.9339	−7.72468	−19.8999	−4.23481
Ellagic acid	−4.2133	4.5235729	14.63135	7.640516	−6.31675	−19.1073	−4.2133
Ellagic acid	−4.11208	3.4813149	14.97426	−26.417	−8.96398	−18.4696	−4.11208
Ellagic acid	−4.08451	2.8578191	14.7638	−21.7358	−6.67745	−16.6032	−4.08451

**Table 6:** Docking scores and energies of chlorogenic acid and ellagic acid with the crystal structure of *G. candidum* (4ZZT).

Mol	S	Rmsd_Refine	E_Conf	E_Place	E_Score1	E_Refine	E_Score2
Chlorogenic acid	−7.84379	1.9669101	0.067377	−99.6069	−12.9229	−50.3995	−7.84379
Chlorogenic acid	−7.25803	1.8288656	12.89607	−99.626	−13.0726	−46.7111	−7.25803
Chlorogenic acid	−7.23898	2.5565467	5.357535	−91.2855	−12.8967	−46.316	−7.23898
Chlorogenic acid	−7.23589	2.2799456	14.8198	−101.412	−13.962	−45.1973	−7.23589
Chlorogenic acid	−7.12938	1.8402419	12.1299	−83.0073	−13.2714	−44.543	−7.12938
Ellagic acid	−6.18615	0.88734156	15.62339	−92.791	−13.3693	−39.0664	−6.18615
Ellagic acid	−6.08951	1.0905997	17.58312	−94.3595	−13.0066	−37.9829	−6.08951
Ellagic acid	−6.08205	1.4268098	15.37853	−85.0092	−12.7316	−33.7174	−6.08205
Ellagic acid	−6.07779	1.5727613	16.8008	−94.8967	−12.7112	−34.4453	−6.07779
Ellagic acid	−6.07731	2.3918681	15.11539	−88.7238	−13.13	−34.386	−6.07731

**Table 7:** Interaction of chlorogenic acid and ellagic acid with the crystal structure of *C. albicans* (4YDE).

Mol	Ligand		Receptor				Interaction	Distance	E (kcal/mol)
Chlorogenic acid	C	26	OD1	ASP	527	(B)	H-donor	3.48	−0.5
	O	40	OD2	ASP	569	(B)	H-donor	2.94	−2.0
	O	23	NZ	LYS	512	(B)	H-acceptor	3.18	−0.8
Ellagic acid	O	20	OD1	ASP	527	(B)	H-donor	2.84	−5.0

**Table 8:** Interaction of chlorogenic acid and ellagic acid with the crystal structure of *G. candidum* (4ZZT).

Mol	Ligand		Receptor				Interaction	Distance	E (kcal/mol)
Chlorogenic acid	O	19	OE2	GLU	217	(A)	H-donor	2.80	−3.8
	O	17	NE2	GLN	175	(A)	H-acceptor	2.97	−1.1
	O	19	NE2	HIS	228	(A)	H-acceptor	3.24	−1.7
	C	1	6-ring	TRP	380	(A)	H-pi	3.91	−0.6
	6-ring		5-ring	TRP	371	(A)	pi-pi	3.61	−0.0
Ellagic acid	O	20	OE2	GLU	217	(A)	H-donor	2.67	−6.2
	O	22	OG1	THR	226	(A)	H-donor	3.20	−1.2
	O	24	NH1	ARG	399	(A)	H-acceptor	3.08	−2.4
	6-ring		NH2	ARG	251	(A)	pi-cation	4.09	−3.2

## Data Availability

Not applicable.
